# Loss of skeletal muscle mass during palliative chemotherapy is a poor prognostic factor in patients with advanced gastric cancer

**DOI:** 10.1038/s41598-020-74765-8

**Published:** 2020-10-19

**Authors:** Song Ee Park, Jin Hwa Choi, Jae Yong Park, Beom Jin Kim, Jae Gyu Kim, Jong Won Kim, Joong-Min Park, Kyong-Choun Chi, In Gyu Hwang

**Affiliations:** 1 Division of Hemato-Oncology, Department of Internal Medicine, Chung-Ang University College of Medicine, 224-1 Heukseok-dong, Dongjak-gu, 06973 Seoul, South Korea; 2 Department of Radiation Oncology, Chung-Ang University Hospital, Seoul, South Korea; 3 Department of Surgery, Chung-Ang University College of Medicine, Seoul, South Korea

**Keywords:** Cancer, Oncology

## Abstract

Cancer causes muscle mass loss, which is associated with a poor prognosis. Chemotherapy may also reduce muscle mass. We investigated skeletal muscle mass change during palliative chemotherapy for advanced gastric cancer (AGC) and its association with treatment outcomes. We retrospectively reviewed 111 consecutive AGC patients who underwent first-line palliative chemotherapy. Skeletal muscle area was measured before and after chemotherapy at the third lumbar vertebra level using computed tomography scans. We compared skeletal muscle index (SMI), body mass index (BMI), and body weight changes to chemotherapy response and survival. The 80 male and 31 female patients’ median age was 65 (range 31–87) years, and 46.8% had sarcopenia at baseline. Median pre-chemotherapy to post-chemotherapy SMI, BMI, and body weight decreases were − 4.5 cm^2^/m^2^ (− 11.3%) (P < 0.001); − 0.7 kg/m^2^ (− 3.2%) (P < 0.001); and − 2.0 kg (− 3.5%) (P < 0.001), respectively. Median SMI decreases for patients with objective response, stable disease, and disease progression were − 4.0 cm^2^/m^2^ (range − 20.1 ~ 9.5); − 4.5 cm^2^/m^2^ (range − 19.8 ~ 0.8); and − 3.8 cm^2^/m^2^ (range: − 17.6 ~ 0.1), respectively. Response to chemotherapy was not associated with SMI decrease (P = 0.463). In multivariable analysis, sarcopenia at baseline (HR 1.681; 95% CI 1.083–2.609, P = 0.021), decreased SMI (HR 1.620; 95% CI 1.041–2.520; P = 0.032) were significant poor prognostic factors for survival. Skeletal muscle mass decreased significantly during chemotherapy in AGC patients, but was not associated with chemotherapy response. Decreased SMI was a poor prognostic factor in AGC patients during first-line palliative chemotherapy.

## Introduction

Advanced gastric cancer (AGC) is the fifth most common cancer in the world and gastric cancer is the third most common cause of cancer related death^1^. The prognosis of patients diagnosed with gastric cancer stage IV is poor. The median overall survival was 11 to 12 months in patients with gastric cancer who received first-line palliative chemotherapy^2,3^.

Cancer cachexia is one of the poor prognostic factors of AGC^4^. Cancer cachexia is a multifactorial syndrome defined by an ongoing loss of skeletal muscle mass that cannot be fully reversed by nutritional support^5^. Skeletal muscle mass depletion is a common feature of cancer cachexia that has been associated with a higher incidence of chemotherapy toxicity, shorter time to disease progression, and shorter survival^4,6^.

Cancer patients have a higher incidence of low skeletal muscle than healthy people, with an incidence rate from 15–40%^7,8^. In patients with gastric cancer, the incidence rate before chemotherapy was reported as 47%^9^. The high prevalence of low skeletal muscle mass in patients with gastric cancer is likely due to poor nutrition, aging and platinum based chemotherapeutic drugs^9–12^. Recently, we also found that sarcopenia (severe low skeletal muscle mass at diagnosis) was an independent prognostic factor in rectal cancer^13^.

Chemotherapy may also reduce skeletal muscle mass^14,15^. Chemotherapeutic agents appear to be taken up by skeletal muscle cells and induce proteolytic and oxidative damage, mitochondrial dysfunction, cellular energy depletion and apoptotic cell death^16,17^. Omega 3 fatty acids have been shown to reduce the muscle loss associated with cisplatin chemotherapy^16^. We hypothesized that not only low skeletal muscle at the time of diagnosis (sarcopenia), but also continued decreases in skeletal muscle mass during chemotherapy would have a prognostic effect on survival. Therefore, we aimed to investigate changes of body composition including weight loss, change of body mass index (BMI) and change of skeletal muscle mass during chemotherapy, and their association with treatment outcomes and survival in patients with advanced gastric cancer (AGC) who received first-line palliative chemotherapy.

## Results

### Patients

From September 2010 through December 2019, 194 patients received chemotherapy for AGC. As of June 2020, 33 patients had disease recurrence during adjuvant chemotherapy or within 6 months after adjuvant chemotherapy, 13 had not received first-line palliative chemotherapy in our hospital, and 37 patients did not have APCT at the time diagnosis of progression. Thus, 111 patients met the inclusion criteria and were included in the analyses. The baseline characteristics of the patients are described in Table 1. The median age was 65 years (range 31–87 years). Among them, 57 (51.4%) patients were over 65 years old and 57 (51.4%) were male. The incidence of sarcopenia at time of diagnosis was 46.8% (52 patients), including 44 men and 8 women. At baseline, half of the patients (54.1%) had normal BMI.

**Table 1:** Baseline characteristics.

Characteristics	Total (N = 111)
**Age—year**	
Median	65
Range	31–87
Age ≥ 65	57 (51.4%)
**ECOG**	
0–1	79 (71.2%)
2	32 (28.8%)
**Sex, n (%)**	
Male	80 (72.1%)
Female	31 (27.9%)
**Metastases**	
1	44 (39.6%)
≥ 2	67 (60.4%)
**Surgery**	
No	72 (64.9%)
Subtotal gastrectomy	22 (19.8%)
Total gastrectomy	17 (15.3%)
Height (m), mean ± SD	162.6 ± 9.0
Weight (kg), mean ± SD	58.2 ± 12.3
**BMI, kg/m**^**2**^	
Underweight < 20	34 (30.6%)
Normal 20–24.9	60 (54.1%)
Overweight > 25	17 (15.3%)
L3 Skeletal muscle index (cm^2^/m^2^)	40.7 ± 9.0
Sarcopenia, n (%)	52 (46.8%)
Hemoglobin (g/dL), median (range)	10.9 (4.0–15.4)
**Serum albumin (g/dL)**	
Median (range)	3.6 (1.5–4.7)
< 3.5 g/dL	45 (40.5%)
**First-line regimen**	
S-1/cisplatin or XP	13 (11.7%)
FOLFOX or XELOX	78 (70.3%)
Trastuzumab plus XP	11 (9.9%)
S-1 or capecitabine	9 (8.1%)

*Sarcopenia* was defined as an L3 skeletal muscle index of < 49 cm^2^/m^2^ for men and < 31 cm^2^/m^2^ for women using Korean-specific cutoffs.

*SMI* skeletal muscle index, *ECOG* Eastern Cooperative Oncology Group, *BMI* body mass index, *FOLFOX* 5-fluorouracil, leucovorin, and oxaliplatin, *XELOX* capecitabine and oxaliplatin, *XP* capecitabine and cisplatin, *ORR* overall response rate.

### Changes in body composition during palliative chemotherapy

The median SMI was 39.6 cm^2^/m^2^ (range 22.0 ~ 67.5 cm^2^/m^2^) pre-chemotherapy and 34.5 cm^2^/m^2^ (range 14.8 ~ 56.9 cm^2^/m^2^) post-chemotherapy, with a median [mean] decrease of − 4.5 [− 5.3] cm^2^/m^2^ (− 11.3% [− 13.0%]) (P < 0.001). The median SMI decrease was − 4.5 cm^2^/m^2^ (range − 20.1 ~ 9.5 cm^2^/m^2^) (− 10.1%) (P < 0.001) for males, and − 4.5 cm^2^/m^2^ (range − 15.0 ~ − 0.2 cm^2^/m^2^) (− 12.8%) (P < 0.001) for females. The median BMI was 21.6 kg/m^2^ (range 14.8 ~ 32.0 kg/m^2^) (− 3.2%) and 34 patients (30.6%) were underweight. The median BMI decrease was − 0.7 kg/m^2^ (range − 5.1 ~ 3.6 kg/m^2^) (− 3.1%) (P < 0.001) for males and − 0.4 kg/m^2^ (range − 4.3 ~ 4.4 kg/m^2^) (− 1.9%) (P = 0.283) for females. The median body weight decrease was − 2.0 kg (range : − 16 ~ 12 kg) (− 3.2%) (P < 0.001) for all patients. The median body weight decreased significantly for males by − 2.0 kg (range : − 16 ~ 10 kg) (− 3.3%) (P < 0.001), but not for females: − 1.0 kg (range : − 10 ~ 12 kg) (− 2.0%) (P = 0.342) (Table 2).

**Table 2:** Changes in body composition during palliative chemotherapy.

	Pre-chemotherapy	Post-chemotherapy	Δ	Percent	P value
Skeletal muscle index , Median [Mean] (range) (cm^2^/m^2^)	39.6 [40.7] (22.0–67.5)	34.5 [35.3] (14.8–56.9)	− 4.5 [− 5.3]	− 11.3% [− 13.0%]	< 0.001
Male	42.2 [42.8] (23.0–67.5)	36.7 [37.6] (17.8–56.9)	− 4.3 [− 5.2]	− 10.1% [− 12.1%]	< 0.001
Female	35.2 [35.1] (22.0–47.2)	29.9 [29.4] (14.8–46.6)	− 4.5 [− 5.7]	− 12.8% [− 16.2%]	< 0.001
BMI, Median [Mean] (range) (kg/m^2^)	21.6 [21.8] (14.8–32.0)	21.1[21.1] (14.8–34.9)	− 0.7 [− 0.7]	− 3.2% [− 3.2%]	< 0.001
Male	21.9 [22.2] (15.2–32.0)	21.3 [21.3] (15.2–34.9)	− 0.7 [− 0.9]	− 3.1% [− 4.0%]	< 0.001
Female	20.8 [20.8] (14.8–31.6)	20.6 [20.5] (14.8–28.2)	− 0.4 [− 0.3]	− 1.9% [− 1.4%]	0.174
Weight, Median [Mean] (range) (kg)	57.0 [58.2] (32.0–95.0)	55.0 [56.1] (32.0–102.0)	− 2.0 [− 2.0]	− 3.5% [− 3.4%]	< 0.001
Male	59.0 [61.5] (39.0–95.0)	57.0 [59.0] (39.0–102.0)	− 2.0 [− 2.5]	− 3.3% [− 4.0%]	< 0.001
Female	49.0 [49.7] (32.0–83.0)	47.0 [48.9] (32.0–74.0)	− 1.0 [− 0.8]	− 2.0% [− 1.6%]	0.142

*BMI* body mass index, *SD* standard deviation.

### Survival

**Figure. 1:**
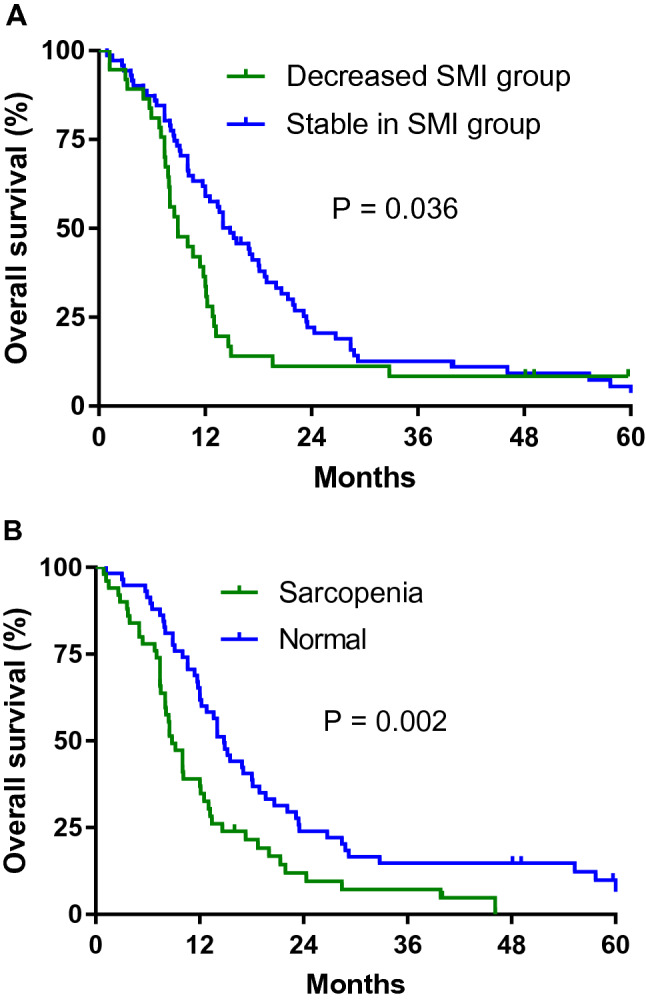
Overall survival according to change in skeletal muscle index (SMI) from pre-chemotherapy to post-chemotherapy (**A**) and sarcopenia (**B**).

The cutoff time for analyses was June 2020, resulting in a median follow-up of 59.7 months (range 0.9–97.2 months), and 98 patients (88.3%) died. For all patients, median OS was 12.1 months (95% CI 10.4–13.7 months). Patients with an SMI decrease > 6.5 cm^2^/m^2^ (− 15.2%) during chemotherapy were defined as the decreased SMI group (n = 39, 35.1%). The remaining 72 patients (64.9%) had maintained SMI. Median PFS was 5.0 months (range 0.4–25.4 months). The decreased SMI group had a significantly shorter OS compared to the maintained SMI group (8.9 months vs. 14.8 months, HR 1.569, 95% CI 1.023–2.407, P = 0.036). The patients with sarcopenia had a significantly shorter OS than patients without sarcopenia (9.2 months vs. 14.8 months, HR 1.865, 95% CI 1.238–2.811, P = 0.002) in gastric cancer (Fig. 1).

In the univariate analysis, sarcopenia (HR 1.865, 95% CI 1.238–2.811, P = 0.003), decreased SMI (HR 1.569, 95% CI 1.023–2.407, P = 0.039), ECOG 2 (HR 1.922, 95% CI 1.212–3.047, P = 0.008), overall response rate (ORR) (HR 0.509, 95% CI 0.322–0.805, P = 0.004) and low albumin (HR 1.592, 95% CI 1.054–2.407, P = 0.027) were significant prognostic factors for survival. In multivariable analysis and sarcopenia at baseline (HR 1.681; 95% CI 1.083–2.609, P = 0.021) and decreased SMI (HR 1.620; 95% CI 1.041–2.520; P = 0.032) were significant poor prognostic factors for survival. Overall response rate (HR 0.603; 95% CI 0.3714–0.978; P = 0.040) was good prognostic factor for survival (Table 3).

**Table 3:** Univariate and multivariate analysis of overall survival.

Variables	Univariate analysis			Multivariate analysis		
	HR	95% CI	P value	HR	95% CI	P value
Age ≥ 65	1.068	0.672–1.698	0.782			
ECOG 2	2.535	1.483–4.332	0.001	1.902	1.081–3.346	0.026
Female sex	1.246	0.749–2.072	0.397			
Metastasis (≥ 2)	1.223	0.768–1.949	0.397			
Surgery	1.203	0.734–1.971	0.463			
Underweight (BMI < 20)	0.8	0.489–1.308	0.369			
Decreased SMI	1.742	1.072–2.833	0.025	1.747	1.042–2.929	0.034
Sarcopenia	1.944	1.214–3.133	0.006	1.959	1.190–3.225	0.008
ORR	0.505	0.299–0.854	0.011	0.684	0.391–1.196	0.183
Hb < 10 g/dL	1.447	0.878–2387	0.157			
Alb < 3.5 g/dL	1.502	0.925–2.437	0.103			

*CI* confidence interval, *HR* hazard ratio, *SMI* skeletal muscle index, *ECOG* Eastern Cooperative Oncology Group, *ORR* overall response rate, *BMI* body mass index, *Hb* hemoglobin, *Alb* Albumin.

### Change in skeletal muscle index in patients according to response to treatment

**Figure. 2:**
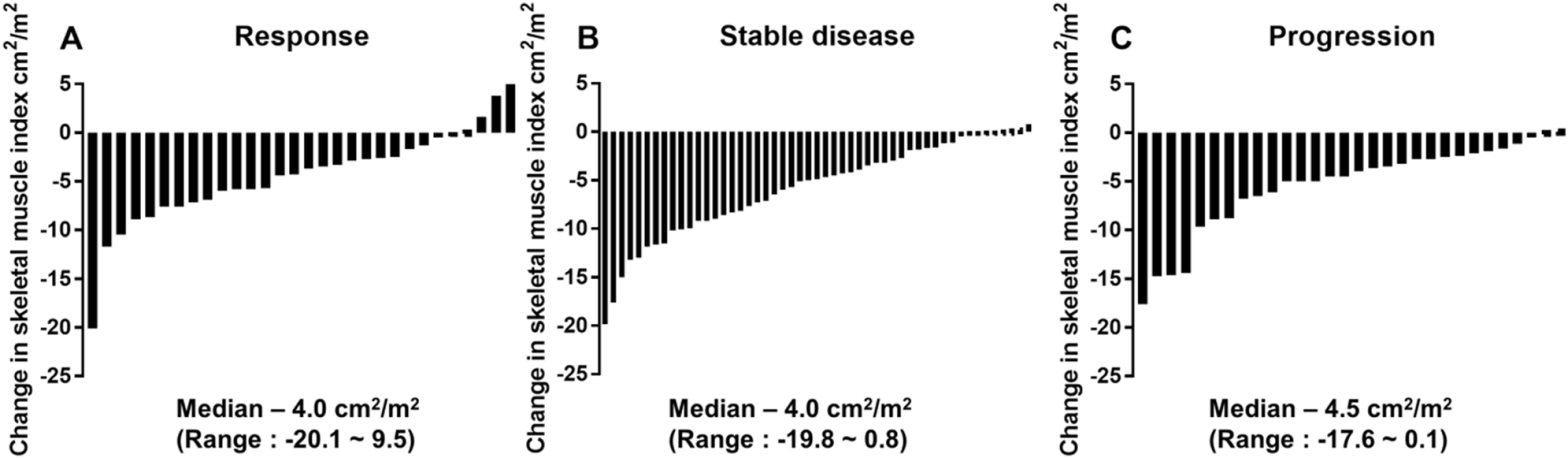
Change in skeletal muscle index according to response to treatment.

The overall response rate during palliative first-line chemotherapy was 27.0% in patients with AGC. Among patients with response, the median SMI decrease was − 4.0 cm^2^/m^2^ (range: − 20.1–9.5 cm^2^/m^2^). This median SMI decrease was − 4.5 cm^2^/m^2^ (range: − 19.8–0.8 cm^2^/m^2^), in those with stable disease and − 3.8 cm^2^/m^2^ (range: − 20.1–9.5 cm^2^/m^2^) in those with progression. The response to chemotherapy was not associated with decrease in skeletal muscle mass (P = 0.463) as illustrated in Fig. 2.

## Discussion

This study presented a decrease in muscle mass during the whole first line chemotherapy period for patients with AGC. We found that decreased SMI during chemotherapy and low skeletal muscle at diagnosis (sarcopenia) were poor prognostic factors in patients with gastric cancer who received palliative first-line chemotherapy. However, a decrease in skeletal muscle mass was not associated with response to chemotherapy. Loss of muscle mass, not weight loss or BMI reduction, was a significant prognostic factor in gastric cancer patients during palliative chemotherapy.

Using baseline CT before palliative chemotherapy and at time of disease progression, we showed decreased skeletal muscle mass (− 11.3%) during chemotherapy in patients with gastric cancer. Similar to our study, decreased SMI during systemic chemotherapy has been found in other studies of colon cancer^14^, NSCLC^15^ and pancreatic cancer^18^. In colon cancer patients, muscle loss was 6.1% during 3 months of chemotherapy^14^. NSCLC patients lost 3.7% SMI during chemotherapy^15^.

Sugiyama et al. also reported that muscle loss during chemotherapy negatively affected survival in gastric cancer patients^19^. Both our study and the Sugiyama et al. study used the approximately lowest tertile as the cut-off value for decrease in SMI. Sugiyama et al. evaluated muscle change for 8 weeks after chemotherapy, while we evaluated muscle change until the time of progression, the whole first line chemotherapy period (median 5.2 months). Therefore, the mean SMI of our study was more reduced than the mean SMI of Sugiyama et al. study (− 13.0% vs. − 7.0%, respectively) and there was a different value of decreased in SMI due to a longer follow up duration in our study (− 15.2% vs. − 10%, respectively). Our results suggested that continuous supportive care for the muscle mass loss were required in gastric cancer patients during the whole first line chemotherapy period. It was suggested that the involvement of digestive organs in gastric cancer makes it more difficult for patients to eat, resulting in a further decrease in muscle mass.

Low skeletal muscle at diagnosis (sarcopenia) is a poor prognostic factor in various cancers^13^. We also found that sarcopenia at baseline was a significant prognostic factor for survival in gastric cancer patients. Using baseline CTs, we showed that sarcopenia is common in patients with gastric cancer (46.8%). This is consistent with another study, where the prevalence of sarcopenia was 47.9% in patients with gastric cancer before palliative first-line chemotherapy and patients with sarcopenia had a significantly shorter OS after chemotherapy^9^.

Sarcopenia was a significantly poor prognostic factor for the decreased SMI group. In our study, we found that an SMI decreased group was associated with significantly shorter OS in patients with gastric cancer. Similarly, a previous study revealed that SMI decreases of more than 9% during chemotherapy were associated with a shorter overall survival in colon cancer^14^. The rate of cancer cachexia in gastric cancer patients was also higher than that in other cancers, and the rate of SMI decrease in gastric cancer was 13.0%. This suggests that we need to improve the prognosis of cancer patients by conducting further studies on various therapies, including exercise and high protein diets to prevent the decreased SMI during chemotherapy.

In our study, SMI, BMI, and body weight were significantly reduced during palliative chemotherapy in gastric cancer. The chemotherapy regimens in metastatic colorectal cancer, involved oxaliplatin, occurred skeletal muscle loss^14,20^. Our studies also suggested that sarcopenia and skeletal muscle loss occurred during chemotherapy because most patients underwent platinum based chemotherapy. Decrease in skeletal muscle mass showed a poor prognostic factor. BMI and body weight change were not significantly poor prognostic factors. Other studies have also shown no prognostic significance for changes in BMI, however, one study reported that an SMI decrease during palliative chemotherapy in biliary cancer was a poor prognostic factor^21^.

In conclusion, the present study showed skeletal muscle mass decreased significantly during chemotherapy in patients with AGC and this skeletal muscle loss during chemotherapy was independently associated with poor survival. However, response to chemotherapy was not associated with decrease in skeletal muscle mass. Further studies are required to improve sarcopenia and muscle loss through interventions such as exercise and high protein diet during chemotherapy and may lead to improved clinical outcomes.

## Methods

### Patients

This retrospective study assessed patients who underwent chemotherapy for gastric cancer in Chung-Ang University College of Medicine between September 2010 and December 2019. The inclusion criteria for analysis were as follows: pathologically confirmed gastric adenocarcinoma; underwent first-line palliative chemotherapy; had enhanced abdominopelvic computed tomography (APCT) within 4 weeks of initiation of first-line chemotherapy and at the time of diagnosis of progression, and an Eastern Cooperative Oncology Group (ECOG) score of 0–2. Exclusion criteria were disease recurrence during adjuvant chemotherapy or within 6 months after adjuvant chemotherapy; palliative first-line chemotherapy not received in Chung-Ang University College of Medicine, and no APCT at the time of diagnosis of progression. This study was approved by the Institutional Review Board of Chung-Ang University Hospital (No. 1909-003-16278). All methods were performed in accordance with the relevant guidelines and regulations. A waiver for the need for informed consent was obtained as part of the IRB approval for this retrospective study.

### Measurements

Skeletal muscle area was measured using APCT scans at baseline (within 4 weeks of initiation of first-line chemotherapy) and at the time of diagnosis of progression. We evaluated skeletal muscle area using the third lumbar vertebra (L3) muscle index, one of the international standards for measuring the ratio of skeletal muscle area (cm^2^) divided by height (m^2^)^22,23^. The cutoff of a decreased SMI was defined approximately lowest tertile according to Blauwhoff-Buskermolen et al. study^14^. We also divided the patients into three groups were categorized into tertiles the muscle change. Therefore, in our study, SMI decreased group was defined a decrease SMI of − 6.5 cm^2^/m^2^ or more based on approximately lowest tertile. Sarcopenia defined as < 31 cm^2^/m^2^ for females and < 49 cm^2^/m^2^ for males on the L3 skeletal index by Korean specific range^13,24,25^. BMI was calculated as weight divided by height squared (kg/m^2^). BMI were divided as underweight (< 20.0 kg/m^2^), normal (20.0–25.0 kg/m^2^), overweight (> 25.0 kg/m^2^)^26^. Body weight during chemotherapy was obtained from medical records within 2 weeks of the date of the APCT. SMI change during palliative chemotherapy was calculated between baseline and the time diagnosis of progression. Clinicopathological data included age, ECOG performance status, sex, height, weight, BMI, SMI, hemoglobin, serum albumin, chemotherapy regimen, chemotherapy response, and survival status. Response evaluation was performed according to the Response Evaluation Criteria in Solid Tumors (RECIST) version 1.1 using follow-up CT obtained every 6 to 8 weeks during chemotherapy. Overall response rate (ORR) was defined as the proportion of patients with tumor size reduction of a predefined amount and for a minimum time period.

### Statistics

Statistical analyses were performed using the Statistical Package for Social Sciences (SPSS) version 24.0 (IBM Corp., Armonk, NY, USA). Statistical significance was defined at P < 0.05. Overall survival (OS) was defined as the time from the first day of treatment to death by any cause. Kaplan–Meier method was used to estimate OS. Hazard ratios (HRs) and their corresponding 95% confidence intervals (CI) were stratified using Cox proportional hazards regression model.

For continuous variables, normality of date was tested using Kolmogorov–Smirnov test. Statistical analyses were performed using Student t test to compare means and the 2-paired sample Wilcoxon signed rank test.

### Ethical approval

This study was approved by the Institutional Review Board of Chung-Ang University Hospital.
